# iStent inject trabecular microbypass stent implantation with cataract extraction in open-angle glaucoma: early clinical experience

**DOI:** 10.1186/s40662-020-00194-3

**Published:** 2020-05-20

**Authors:** Tanner J. Ferguson, Zachary Dockter, Adam Bleeker, Kayla L. Karpuk, Justin Schweitzer, Mitch J. Ibach, John P. Berdahl

**Affiliations:** 1 Cole Eye Institute, Cleveland Clinic, Cleveland, OH USA; 2 University of South Dakota Sanford School of Medicine, Sioux Falls, SD USA; 3 Cleveland Eye Clinic, Cleveland, OH USA; 4 Vance Thompson Vision, Sioux Falls, SD USA

**Keywords:** Micro-invasive glaucoma surgery, Minimally invasive glaucoma surgery, MIGS, Trabecular microbypass stent, Open-angle glaucoma

## Abstract

**Background:**

Retrospective, consecutive case series to evaluate the implantation of two second-generation trabecular microbypass stents in combination with cataract surgery in a real-world, clinical setting.

**Methods:**

The series included 56 eyes implanted with the iStent inject device with phacoemulsification. The series consisted of eyes with primary open-angle glaucoma (*n* = 52) and pseudoexfoliative glaucoma (*n* = 4). Primary outcome measures included intraocular pressure (IOP) and number of glaucoma medications. Safety outcomes included the need for secondary surgical intervention and the incidence of IOP spikes ≥10 mmHg and ≥ 15 mmHg.

**Results:**

IOP was reduced by 21% to 14.7 ± 2.9 mmHg (*p* < 0.01) at 6 months postoperative from 18.7 ± 5.8 mmHg at baseline. Preoperatively, the mean number of glaucoma medications was 1.5 ± 0.9 and reduced by 39% to 0.9 ± 1.2 (*p* < 0.01) at 6 months. At 6 months, 68% of eyes had an IOP ≤15 mmHg, increased from 30% at baseline. 55% of eyes were medication-free at 6 months, up from 18% at baseline. There were no severe postoperative complications. No eyes underwent an additional glaucoma procedure.

**Conclusions:**

Implantation of the iStent inject device with concomitant cataract surgery effectively provides a sustained reduction in IOP with a markedly improved medication burden out to 6 months postoperative. The safety profile is excellent.

## Background

Open-angle glaucoma, a chronic, debilitating disease for patients, remains a leading cause of global blindness [1]. The mainstay of treatment in open-angle glaucoma targets the reduction of intraocular pressure (IOP), the sole modifiable risk factor associated with the disease [2]. Within the last decade, the treatment approach to open-angle glaucoma has undergone notable innovation with the advent of minimally invasive glaucoma surgery (MIGS) [3, 4]. Despite not offering the robust IOP reduction observed with traditional, filtering procedures, this growing class of procedures offers a superior safety profile and preserves the option for additional surgery with most of the surgeries in this class sparing the conjunctiva. The growing lineup of MIGS procedures target IOP reduction via enhanced trabecular outflow, enhanced uveoscleral outflow, enhanced subconjunctival outflow or decreased aqueous production [3]. In addition, several recently introduced options within the MIGS armamentarium target multiple mechanisms for IOP reduction [5]. The first FDA-approved MIGS device, the iStent (Glaukos Corp.), is a trabecular microbypass stent that bypasses the trabecular meshwork to improve physiologic outflow and lower IOP [6]. The iStent device is well studied and established as a safe, effective option for patients with open-angle glaucoma, with and without concomitant cataract surgery [7–9]. Prior reports have also demonstrated that insertion of multiple stents can provide additional IOP reduction [10].

Recently, a second-generation iteration of the iStent was introduced, the iStent inject (Glaukos Corp.), which received FDA approval in 2018. The second-generation device is pre-loaded with two smaller stents for insertion and was created with 4 lateral outlet lumens on each stent allowing for multi-directional outflow in Schlemm’s canal to assess more collector channels [11]. The second-generation iStent inject was designed to provide further IOP reduction beyond the first-generation device, which would be expected based on clinical studies demonstrating the additional IOP reduction achieved with multiple insertion of the first-generation iStent [10]. In addition, the iStent inject was also engineered with enhanced procedural efficiency in the hopes of mitigating the learning curve for successful implantation. Thus far, studies performed outside the United States evaluating iStent inject have been favorable including multiple reports demonstrating superior IOP reduction accompanied by a reduced medication burden with the iStent inject compared to the first generation iStent [12, 13].

This goal of this study was to evaluate our initial clinical experience with the use of the second-generation device, iStent inject, in combination with cataract surgery. Although the studies performed thus far investigating the iStent inject have largely reported favorable results [14, 15], .this study aims to provide additional results in a real-world setting from an experienced surgeon’s early experience with the second-generation device in combination with cataract surgery. To collect data, we performed a retrospective case review of our initial cases of two second-generation trabecular microbypass stents in combination with cataract surgery.

## Materials and methods

### Study design

This study did not restrict patients to the conventional inclusion and exclusion criteria of a multicenter randomized, controlled clinical trial in an attempt to best simulate the intended clinical use of the device. Therefore, this study did not screen for disease severity, preoperative IOP, or medication use to mimic a real-world, clinical population. This report included eyes that underwent combined cataract surgery with implantation of an iStent inject, a device that encompasses two, biocompatible trabecular micro-bypass stents preloaded on a single injector. Data collected occurred between August 2018 and May 2019, the period when the device initially became available in the US.

The present retrospective, consecutive case series included 56 eyes with open-angle glaucoma, ranging from mild to severe. Notably, the iStent inject device was FDA-approved for mild-moderate stage of open-angle glaucoma with cataract surgery and thus including eyes with severe stage of disease is considered an “off label” use of the device. A consistent cohort of eyes with postoperative data available 6 months after surgery was also established. There were no exclusion criteria. Patients in the study had a preoperative diagnosis of open-angle glaucoma ranging from mild to severe as defined by the American Academy of Ophthalmology’s Preferred Practice Pattern Guidelines, which is primarily based on visual field criteria [16]. Data was collected and evaluated from procedures performed by a single, fellowship-trained surgeon (J.P.B) at a single site (Sioux Falls, SD). This study was approved by the Institutional Review Board at the University of South Dakota and procedures conducted were in accordance with the 1964 Helsinki declaration and its later amendments or comparable ethical standards. This was a retrospective analysis based on information collected from patients’ medical record and all information was de-identified. Thus, the informed consent process was waived by the IRB and was unnecessary.

### Device description, surgical technique

The iStent inject® trabecular micro-bypass stent system includes two stents per device. The two stents are pre-loaded on a single injector that allows for insertion and subsequent bypass in two distinct regions of the trabecular meshwork with a single procedure. In contrast to the prior generation (iStent®), the stents included with the second-generation device are slightly smaller and each stent includes 4 lateral outflow lumens that aim to produce multi-directional outflow and enhanced access to downstream collector channels.

In this study, implantation of the iStent inject occurred following standard phacoemulsification and insertion of IOL (intraocular lens). After cataract removal and IOL insertion, the eye was left dilated with the cohesive ophthalmic viscosurgical device. After rotation of both the patient and the microscope, a gonioprism was gently placed on the cornea with the non-dominant hand. With the gonioprism comfortably placed for adequate visualization, the single-use injector device was inserted through a temporal clear corneal incision and two pre-loaded stents were implanted into the nasal region of Schlemm’s canal approximately 2–3 clock hours apart.

### Postoperative medications

Postoperatively, patients were prescribed moxifloxacin 0.05% for 1 week, daily NSAID (bromfenac 0.07% or nepafenac 0.3%) for 4 weeks and steroid drops (prednisolone acetate 1% or loteprednol etabonate 0.5%) for 4 weeks which were started as 4 times daily and then tapered to 2 times daily after 1 week. All preoperative glaucoma medications were continued for at least 1 week postoperatively and in patients with well-controlled IOP values postoperatively, medications were removed systemically in which a single drop was discontinued and patients were monitored closely for IOP spikes. As opposed to the randomized, controlled clinical trials conducted for various MIGS devices for FDA approval, no washout period was used in this study and decision-making pertaining to the addition/removal of glaucoma medications were based on clinical judgment.

Preoperative data was used to create a baseline, which typically occurred in the visits immediately leading up to the surgical procedure. Postoperatively, data was collected at 1 day, 1 week and months 1, 3 and 6. At each postoperative time point, the recorded data included IOP and the number and type of glaucoma medications.

### Outcome measures and safety evaluation

Primary outcomes for the study were IOP and number of ocular hypotensive medications. The baseline IOP consisted of two measurements obtained via Goldmann applanation tonometry in the two visits leading up to surgery. All combination glaucoma drops were logged as two medications in the data. The incidence of postoperative IOP spikes ≥10 mmHg and ≥ 15 mmHg above baseline, intraoperative and postoperative adverse events, and secondary surgical intervention were recorded for establishment of an appropriate safety profile. Best-corrected visual acuity (BCVA), obtained using a standard Snellen chart, was also collected as part of the safety profile. If an eye underwent a secondary procedure, it was included in the data set until the point of additional surgical intervention.

### Statistical analysis

A paired t test procedure was employed to determine the significance of the mean change in IOP from baseline to the following timepoints: 1, 3, and 6 months. A paired t-test was also used to analyze the mean change in the number of glaucoma medications used at baseline in comparison to 1, 3 and 6 months. All the statistical analyses in this study were performed using SAS software (version 9.4, SAS Institute, Inc.). The significance level was set at 0.05.

## Results

This was a retrospective case series that included 56 eyes from 38 patients from a single center. The average age of the cohort was 71.0 ± 7.7 years; 18 of the 56 eyes were from male patients. 5 of the 56 eyes included in the study had underwent prior selective laser trabeculoplasty treatment. These parameters, in addition to other pre-operative characteristics, are shown in Table 1.

**Table 1:** Demographic and preoperative characteristics

Parameter	Preoperative
Age, years (mean ± SD)	71.0 ± 7.7
Gender (F/M)	38/18
Race/ethnicity	White (100%)
**No. of medications**	
Mean ± SD	1.5 ± 1.1
No. on 0 meds	10 (18%)
No. on 1 meds	23 (41%)
No. on 2 meds	11 (20%)
No. on ≥3 meds	12 (21%)
**Glaucoma type (n)**	
POAG	52
PXG	4
**Glaucoma severity (n)**	
Mild	26
Moderate	26
Severe	4

*OAG* = Open-angle glaucoma; *PXG* = Pseudoexfoliative glaucoma

### Efficacy

**Fig. 1:**
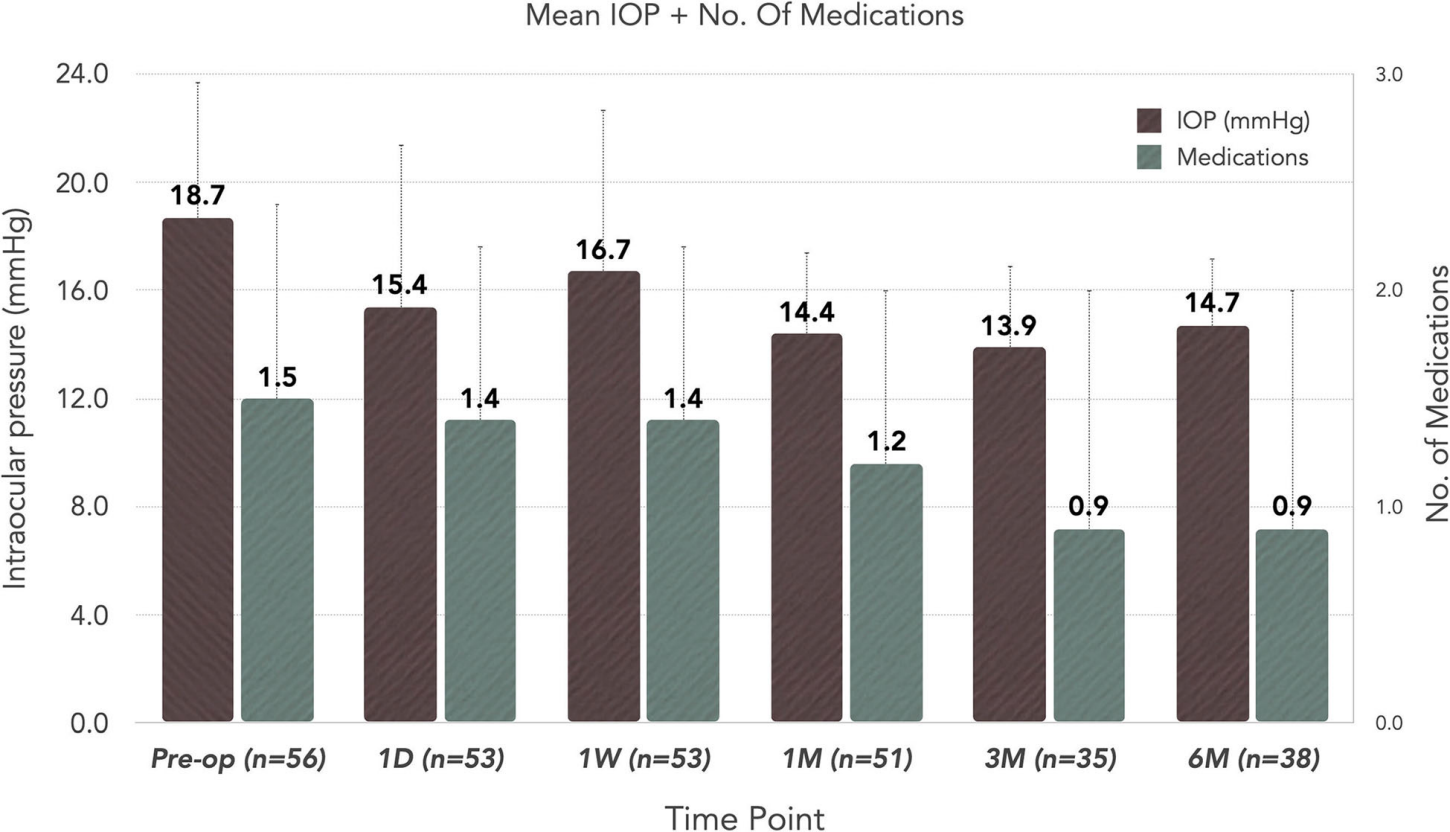
All Eyes -- Mean IOP and Number of Glaucoma Medications. This figure depicts the mean IOP and number of glaucoma medications for all eyes included in the study out to 6 months postoperative. The error bars represent standard deviation

Figure 1 demonstrates the primary outcome measures of the study: mean number of ocular hypotensive medications and mean IOP at each time point. Preoperatively, the baseline mean IOP was 18.7 ± 5.8 mmHg. At the 1-month time point, the mean IOP was 14.4 ± 3.1 mmHg (*p* < 0.01) and this reduction was maintained < 15 mmHg out to 6 months with a mean value of 14.7 ± 2.9 mmHg (*p* < 0.01), indicating a 4 mmHg (21%) reduction in pressure.

**Fig. 2:**
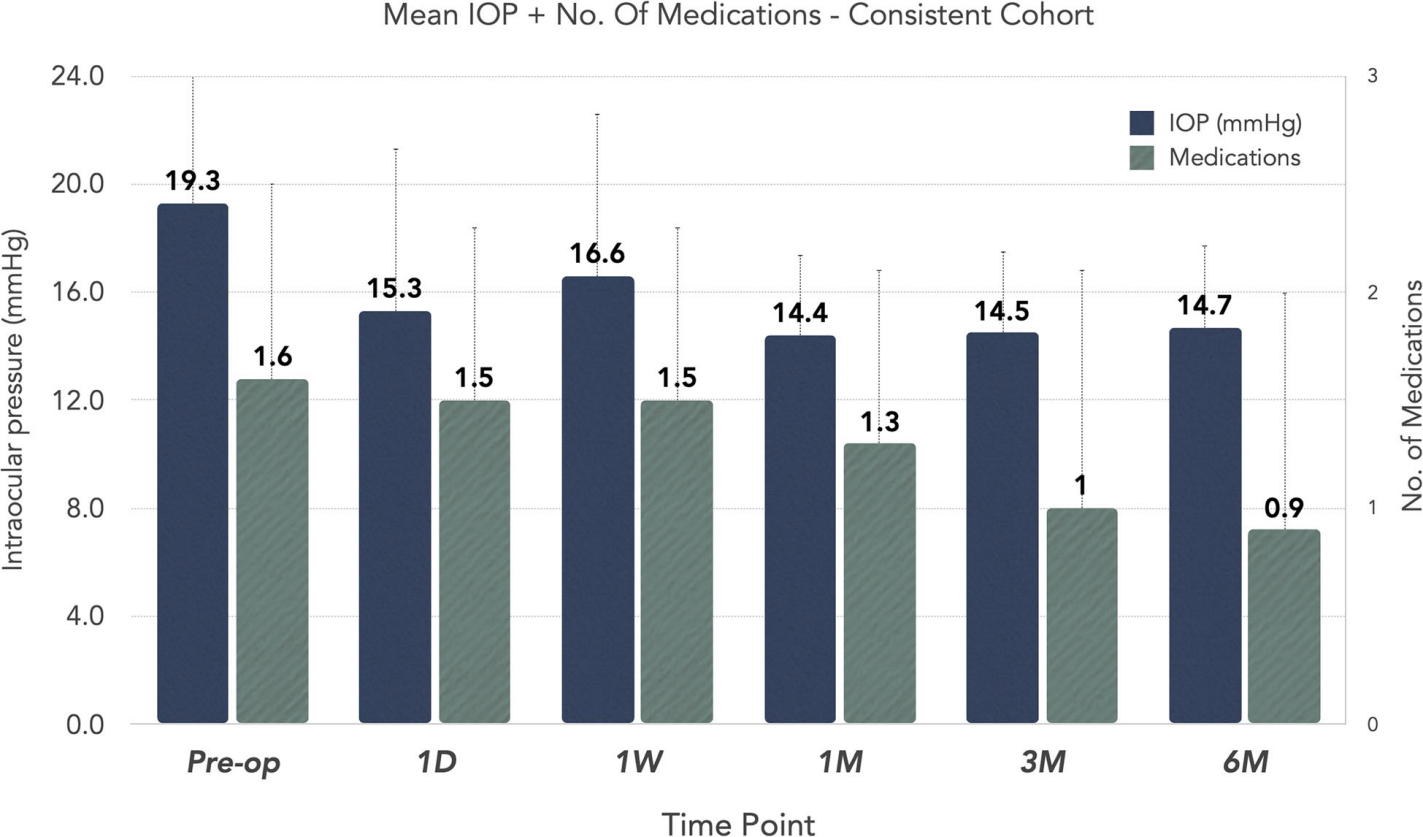
Consistent Cohort -- Mean IOP and Number of Glaucoma Medications. The mean IOP and number of glaucoma medications is shown for eyes in the established consistent cohort. The consistent cohort is composed of eyes with 6-month data available and was established to directly compare this subset to baseline. The error bars represent standard deviation

A consistent cohort was also created to directly compare eyes with 6-month postoperative data available to baseline. In this cohort (*n* = 38), at baseline, the mean IOP was 19.3 ± 6.3 mmHg and the mean number of medications was 1.6 ± 0.9. At 3 months postoperative, the mean IOP was 14.5 ± 3.0 mmHg and at 6 months, the mean IOP remained < 15 mmHg (14.7 ± 2.9 mmHg), representing a 24% pressure reduction. For medication use, the mean number of medications was reduced by 44% to 0.9 ± 1.2 (*p* < 0.01) from baseline. Figure 2 demonstrates the results of the consistent cohort.

**Fig. 3:**
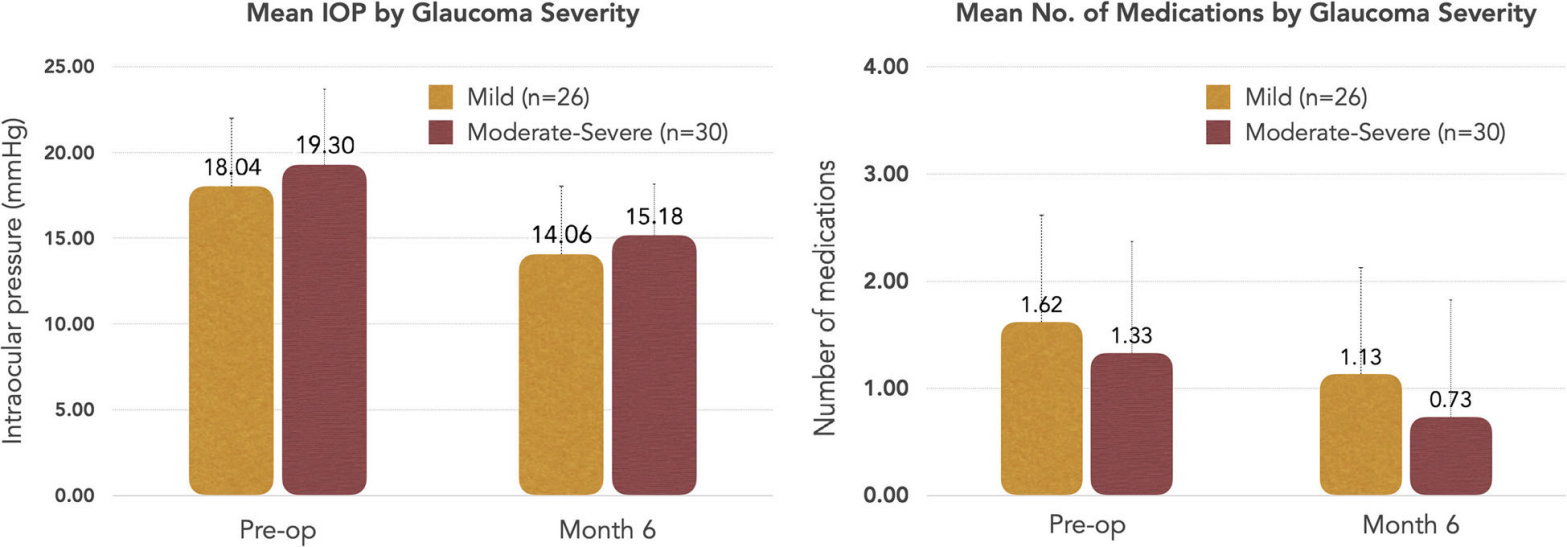
Mean IOP and Number of Glaucoma Medicaitons Stratified by Glaucoma Severity. This figure demonstrates the mean IOP and number of glaucoma medications stratified by glaucoma severity at baseline and at 6 months postoperative. The graph on the left compares mean IOP and the graph on the right compares the mean number of glaucoma medications

This study also stratified results based on severity of disease. Since there were only 3 eyes included in the study with severe stage of glaucoma, these cases were combined with moderate stage of disease for comparing the data. In eyes with mild (*n* = 26) stage of disease, the baseline mean IOP was 18.0 ± 4.3 mmHg and the mean number of medications was 1.6 ± 1.0. At 6 months postoperative, the mean IOP was reduced to 14.1 ± 2.3 mmHg and the medication burden was decreased to 1.1 ± 1.2 medications. In eyes with moderate-severe (*n* = 30) stage of disease, the mean IOP prior to the surgery was 19.3 ± 6.8 mmHg and baseline mean number of medications was 1.33 ± 1.1. At 6 months postoperative, medication use was reduced 0.7 ± 1.2 and the mean IOP was decreased to 15.2 ± 3.2 mmHg. These results are shown in Fig. 3.

**Fig. 4:**
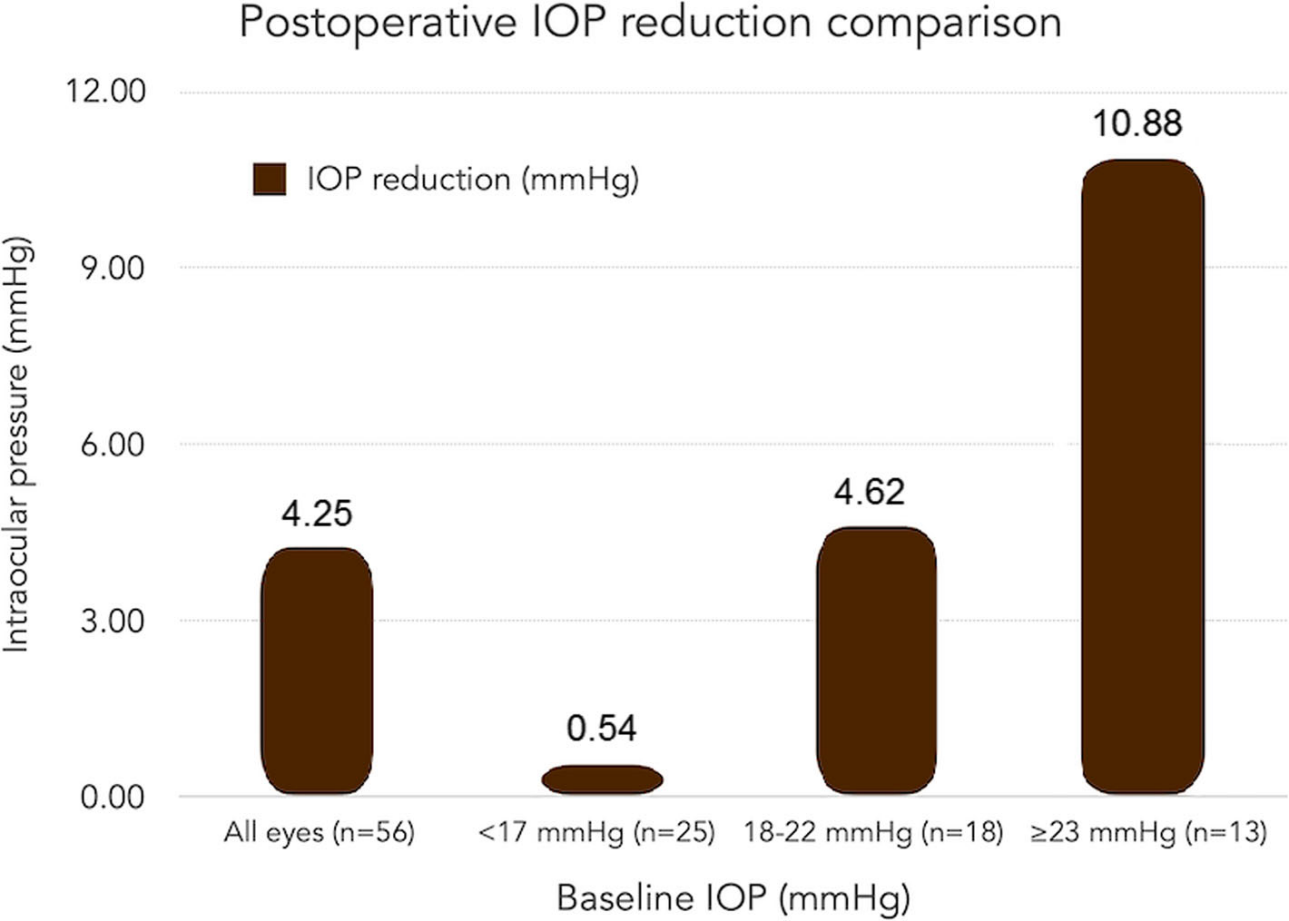
Mean Postoperative IOP Reduction Stratified by Baseline IOP. Mean IOP reduction based on baseline IOP. The bar on the far left represents the mean IOP reduction for each eye’s last collected follow-up IOP value

The magnitude of IOP reduction was also evaluated based on baseline IOP. To compare the level of IOP reduction based on preoperative IOP, the last collected follow-up IOP measurement available (e.g., 6 month) was logged and compared to baseline. Mean follow-up length was 4.7 ± 2.0 months amongst the 56 eyes included in this study. In patients with baseline IOP < 17 mmHg, the mean reduction in IOP was < 1 mmHg (0.5 ± 3.7 mmHg). Patients with preoperative IOP ranging from 17 to 22 mmHg had a mean reduction in IOP of 4.6 ± 2.4 mmHg and patients with preoperative IOP measurement > 22 mmHg achieved a mean reduction of 10.9 ± 4.7 mmHg at their last collected follow up. Figure 4 shows these results.

**Fig. 5:**
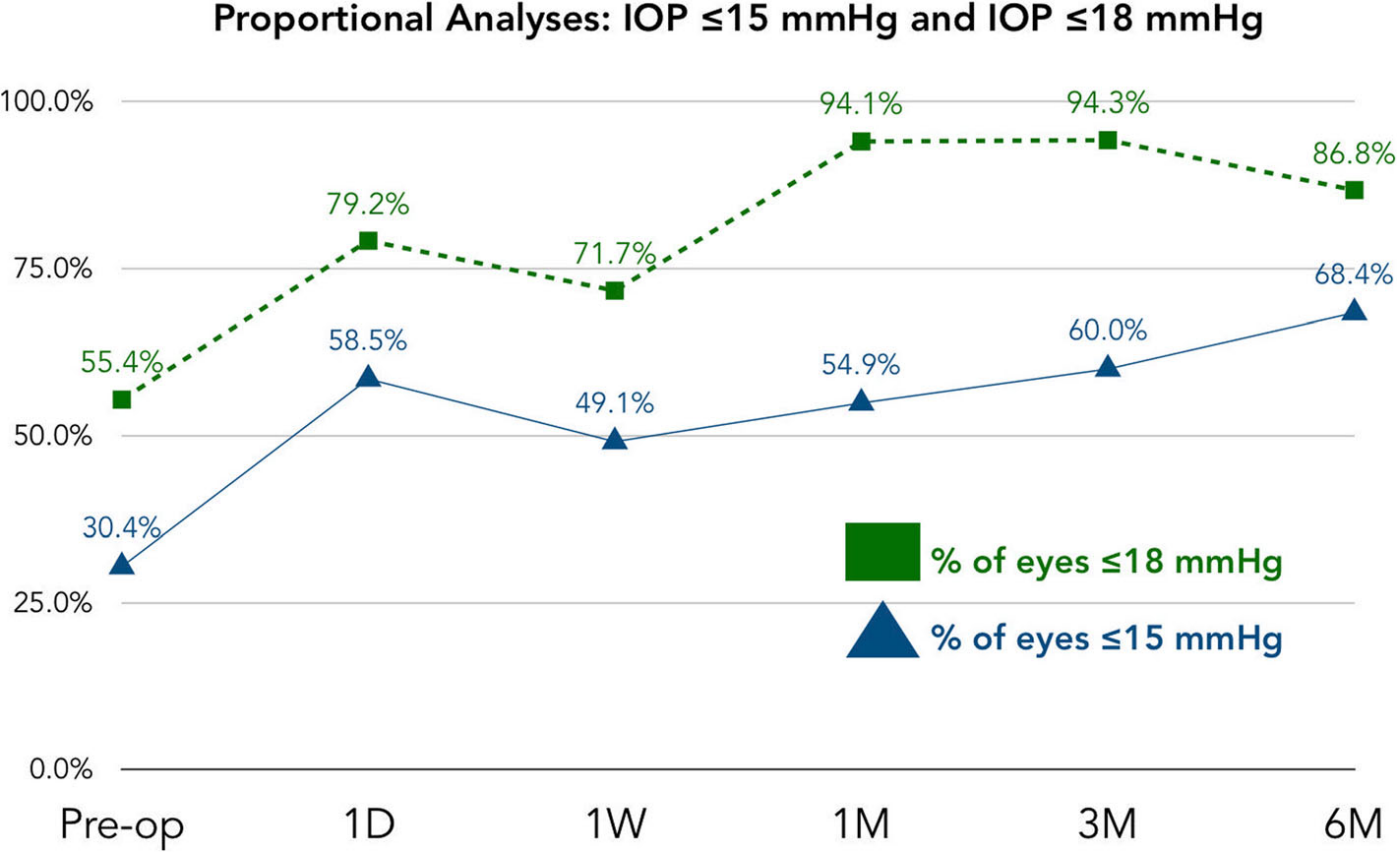
Proportional IOP Analyses. This figure demonstrates the IOP proportional analyses including the percentage of eyes at each time point with IOP ≤15 mmHg and IOP ≤18 mmHg

This study also included proportional IOP analyses to compare the number of eyes achieving IOP ≤18 mmHg and ≤ 15 mmHg postoperatively in comparison to baseline. At baseline, 55% (*n* = 31) of eyes were ≤ 18 mmHg and 30% (*n* = 17) of eyes were ≤ 15 mmHg. At 1 month postoperative, the percentage of eyes ≤18 mmHg had increased to 94% and at 6 months, 87% (*n* = 33) of eyes were ≤ 18 mmHg. The percentage of eyes achieving ≤15 mmHg increased to 55% (*n* = 28) at 1 month and was up to 68% (*n* = 26) by 6 months postoperative. These results are shown in Fig. 5.

For medication use, at baseline, the mean number of medications was 1.5 ± 1.0. At baseline, prior to the surgery, 82% (*n* = 46) of eyes were on at least 1 medication with 41% (*n* = 15) of eyes on ≥2 medications. At 1 month postoperative, medication use was not clinically significantly different from baseline with a mean of 1.2 ± 1.0. By 6 months postoperative, medications were reduced by 39% to a mean of 0.9 ± 1.2 medications (*p* < 0.01). At the 6-month postoperative time point, 55% of eyes were medication free, increased from 18% at baseline. In addition, 45% of eyes were on ≥1 medication(s) at 6 months compared to 82% at baseline. For medication reduction, 67% of eyes that were on medication(s) at baseline achieved a reduction in medication use at the 6-month postoperative time point.

### Safety profile

No cases of hypotony (< 6 mmHg) occurred postoperatively. At the 6-month time point, none of the 56 eyes had undergone a secondary glaucoma procedure. There were no intraoperative or postoperative complications related to the surgery. For IOP spikes above baseline, there was only a single case of an IOP increase ≥15 mmHg above baseline and 3 (5%) cases had IOP increases ≥10 mmHg above baseline. There were no sequelae related to the pressure spikes and all occurred within the first week of the postoperative period and responded to topical therapy. All eyes improved or maintained their vision at the 6-month time point with 100% of eyes included in the study achieving a BCVA of 20/30 or better.

## Discussion

Numerous studies have independently evaluated the iStent and iStent inject and established the role of each device, with and without concomitant cataract surgery, in the treatment of open-angle glaucoma. Several clinical studies have been published supporting the use of the iStent as a sole procedure [7, 17]. in combination with other MIGS procedures [18] as well as the implantation of multiple stents [8, 19]. Moreover, there are numerous published studies evaluating the iStent inject that have reported clinically significant long-term reductions in both medication and IOP, with and without cataract surgery [14, 20]. Despite the meaningful data published thus far, to our knowledge, there has been no published experience from the United States with the second-generation iStent inject device since its FDA approval in 2018.

This single-surgeon study provides data from an experienced MIGS surgeon’s early experience with the second-generation device. At 6 months postoperative, there was a greater than 20% reduction in IOP to 14.7 ± 2.9 mmHg from a mean of 19.2 ± 6.3 mmHg at baseline. The percentage of eyes with IOP ≤15 mmHg increased to 68% at 6 months postoperative in comparison to only 30% at baseline. This study also showed a correlation between the magnitude of IOP reduction and baseline IOP, consistent with what has been demonstrated in numerous previous studies related to both MIGS and phacoemulsification alone [7, 21, 22]. In this study, the mean IOP reduction in eyes with a baseline IOP of 18–22 mmHg was 4.6 ± 2.4 mmHg, significantly greater than what was demonstrated in eyes with a baseline IOP ≤17 mmHg (0.5 ± 3.7 mmHg).

In regard to medication use, 82% of eyes were on ≥1 medication(s) at baseline but this was reduced to 45% at 6 months, highlighting the reduced medication burden for patients through 6 months. More importantly, 55% of patients were medication-free at 6 months postoperative in comparison to only 18% prior to the procedure. The benefits of a reduced medication burden are well established and 66% of patients on medications at baseline achieved a reduction in medication use at 6 months. Prior studies have demonstrated that more than 1 topical agent is associated with worse patient adherence [23] and in this study, the percentage of eyes on more than 2 medications was reduced to 26% at 6 months from 41% at baseline. Moreover, with the considerable amount of evidence showing the toxic and inflammatory effect on the ocular surface from glaucoma topical agents, reducing and/or eliminating medications altogether is particularly advantageous [24].

The safety profile in this study was remarkable, consistent with prior studies evaluating the first and second-generation device (iStent and iStent inject, respectively) [25–27]. There were no cases of hypotony. Postoperative IOP spikes ≥15 mmHg and ≥ 10 mmHg were minimal with only a single case of a pressure spike ≥15 mmHg above baseline that occurred within the 1st postoperative week. However, it should be noted that patients were continued on their preoperative glaucoma medications until 1 week, which may have mitigated any post-operative IOP spikes in the first week postoperatively. In addition, no eyes underwent an additional procedure through 6 months postoperative.

The data from this present study comes from a single surgeon (J.P.B.) with extensive experience with the first-generation iStent device [7, 28, 29] ,which likely mitigated any early learning curve with the second-generation device. While it cannot be directly compared, the IOP-lowering results at 6 months in this present study are consistent or superior to what was reported with our experience in eyes with OAG with the first-generation device in combination with cataract surgery, which demonstrated a mean reduction of 3.7 mmHg at 6 months postoperative in the consistent cohort [30], compared to the 4.6 mmHg reduction noted in this study’s consistent cohort. While both technologies demonstrated favorable IOP-lowering results, early data favors the results of the present study evaluating the iStent inject. Nonetheless, this project is ongoing and continued data collection to assess whether the two-stent approach of the iStent inject accessing a broader range of the conventional outflow system will translate to a more sustainable reduction in medication use and IOP in the long-term. Theoretically, a two-stent approach could increase the likelihood of avoiding a region of obstruction downstream and successful bypass of the trabecular meshwork. Future and ongoing studies investigating the long-term safety and efficacy of the iStent inject in comparison to the first iteration of the device will be important for assessing the sustainability of the efficacy observed through 6 months.

This study is not without limitations. It was an open-label and nonrandomized study with no control group. No specific guidelines were employed to direct the decision to add/remove topical medications in the postoperative period. The 18 cases of bilateral implantation could contribute additional bias and is an acknowledged limitation. Moreover, the retrospective design of the study prevented uniform follow-up, which contributes to missing follow-up data at postoperative time points. Another limitation may be selection bias; for example, patient who we believed required immediate IOP reduction to the low teens or single digits selected for a filtering procedure. Despite its limitations, this study population represents a real-world clinician’s glaucoma population and provides insight on the early safety and efficacy of implantation of the iStent inject trabecular microbypass stent.

Future and ongoing studies will be incredibly important for defining the safety profile and evaluating the efficacy of the device in comparison to not only the first-generation device, but other MIGS devices that target the same anatomical space [31]. Furthermore, given the success of the first-generation device in eyes with secondary forms of OAG [22, 28], .studies primarily focused on evaluating the second-generation device in these populations would be valuable. Early long-term results from studies performed outside of the U.S. have been favorable including a study by Hengerer et al. demonstrating a more than 10 mmHg IOP reduction from baseline 3 years postoperatively accompanied by an impressive reduction in medication use [14]. Moreover, comparative studies have demonstrated superior efficacy with the second-generation device in comparison to the first-generation iteration with a similar safety profile [12, 13]. Additional data collection and studies will ultimately be important for validating these early findings, but the results thus far are promising.

## Conclusions

In conclusion, to our knowledge, this is the first real-world clinical study to provide results evaluating the iStent inject in combination with phacoemulsification from a U.S.-based surgeon’s practice. In this study, outcomes through 6 months were favorable with a significant reduction of IOP and medication use accompanied by an excellent safety profile. Moreover, these results did not utilize any strict inclusion criteria regarding baseline IOP and/or medication use and were observed within a realistic clinical setting. Continued and long-term data will be valuable for further evaluating the device but the early results are compelling and support continued use of the device in combination with cataract surgery as a treatment for open-angle glaucoma.

## Data Availability

The datasets used and/or analyzed during the current study are available from the corresponding author on reasonable request.
